# Comparative analysis of the silk gland transcriptomes between the domestic and wild silkworms

**DOI:** 10.1186/s12864-015-1287-9

**Published:** 2015-02-06

**Authors:** Shou-Min Fang, Bi-Li Hu, Qiu-Zhong Zhou, Quan-You Yu, Ze Zhang

**Affiliations:** School of Life Sciences, Chongqing University, Chongqing, 400044 China; Key Sericultural Laboratory of Shaanxi, Ankang University, Ankang, 725000 China; College of Life Science, China West Normal University, Nanchong, 637002 China

**Keywords:** *Bombyx mori*, Domestication, Silk gland, RNA-seq, Differentially expressed gene

## Abstract

**Background:**

*Bombyx mori* was domesticated from the Chinese wild silkworm, *Bombyx mandarina*. Wild and domestic silkworms are good models in which to investigate genes related to silk protein synthesis that may be differentially expressed in silk glands, because their silk productions are very different. Here we used the mRNA deep sequencing (RNA-seq) approach to identify the differentially expressed genes (DEGs) in the transcriptomes of the median/posterior silk glands of two domestic and two wild silkworms.

**Results:**

The results indicated that about 58% of the total genes were expressed (reads per kilo bases per million reads (RPKM) ≥ 1) in each silkworm. Comparisons of the domestic and wild silkworm transcriptomes revealed 32 DEGs, of which 16 were up-regulated in the domestic silkworms compared with in the wild silkworms, and the other 16 were up-regulated in the wild silkworms compared with in the domestic silkworms. Quantitative real-time polymerase chain reaction (qPCR) was performed for 15 randomly selected DEGs in domestic versus wild silkworms. The qPCR results were mostly consistent with the expression levels determined from the RNA-seq data. Based on a Gene Ontology (GO) enrichment analysis and manual annotation, five of the up-regulated DEGs in the wild silkworms were predicted to be involved in immune response, and seven of the up-regulated DEGs were related to the GO term “oxidoreductase activity”, which is associated with antioxidant systems. In the domestic silkworms, the up-regulated DEGs were related mainly to tissue development, secretion of proteins and metabolism.

**Conclusions:**

The up-regulated DEGs in the two domestic silkworms may be involved mainly in the highly efficient biosynthesis and secretion of silk proteins, while the up-regulated DEGs in the two wild silkworms may play more important roles in tolerance to pathogens and environment adaptation. Our results provide a foundation for understanding the molecular mechanisms of the silk production difference between domestic and wild silkworms.

**Electronic supplementary material:**

The online version of this article (doi:10.1186/s12864-015-1287-9) contains supplementary material, which is available to authorized users.

## Background

The domestic silkworm, *Bombyx mori* (*B. mori*), is an economically important insect in countries such as China, Brazil and India. For thousands of years, silk produced by domestic silkworms has been a widely sought fair textile material. *B. mori* was domesticated from the Chinese wild silkworm, *Bombyx mandarina*, approximately 5000 years ago [1-3]. Long-term artificial breeding and selection have resulted in high silk yield in domestic silkworms compared with the silk yield in its wild relative [2,4]. However, little is known about the genetic mechanisms of increased silk yield in the domestic silkworm [5].

The silk proteins (fibroins and sericins) are produced by silk glands. The gland is divided into three compartments: anterior silk gland (ASG), median silk gland (MSG), and posterior silk gland (PSG). ASGs serve as ducts to transport the silk proteins in the final secretion process [6]. PSGs are responsible for the synthesis and secretion of fibroins, which are composed of a heavy chain (Fib-H, molecular weight 391 kDa) [7], a light chain (Fib-L, 26 kDa), and P25 (30 kDa) with a molar ratio of 6:6:1 [8]. MSGs synthesize the glue proteins (sericins), which are composed of seven major sericins that are encoded by three main genes, *Ser1*, *Ser2*, and *Ser3* [9]. Thus, silk proteins are biosynthesized in the MSGs and PSGs. Silk threads comprise approximately 75% insoluble fibrous proteins in the inner layer and 25% hydrophilic glue sericin proteins in the outer layer [6].

To understand the tissue specificity and functional diversification, the silk gland transcriptome in domestic silkworm has been investigated in previous studies [10-13]. Expressed sequence tags were sequenced in the PSGs on days 1 and 5 of fifth-instar larvae [10]. Differential gene expression patterns between anterior/median silk glands (A/MSG) and PSGs were examined on day 3 of fifth-instar larvae [11]. Based on serial analysis of gene expression (SAGE)-aided transcriptome analysis, the gene expression profiles of MSGs and PSGs have also been compared [13]. These studies suggested that the expression levels of most of the protein-coding genes were similar in the MSGs and PSGs of the domestic silkworm, while, as expected, mRNAs encoding the fibroins and sericins showed differential expression patterns.

Previous studies have focused mainly on the silk gland transcriptome in domestic silkworms. However, there seems to be no study on the silk gland transcriptome in *B. mandarina*, the wild relative of the domestic silkworm. Here, we sequenced the transcriptomes of silk glands from day 3 of fifth-instar larvae of two domestic and two wild silkworms using the Illumina sequencing technology. The goal of this study was to identify differentially expressed genes (DEGs) in domestic versus wild silkworms, to help understand the molecular mechanism associated with the difference in silk production between domestic silkworm and its wild ancestor *B. mandarina*.

## Results

### RNA-sequencing and identification of novel transcripts

**Figure 1:**
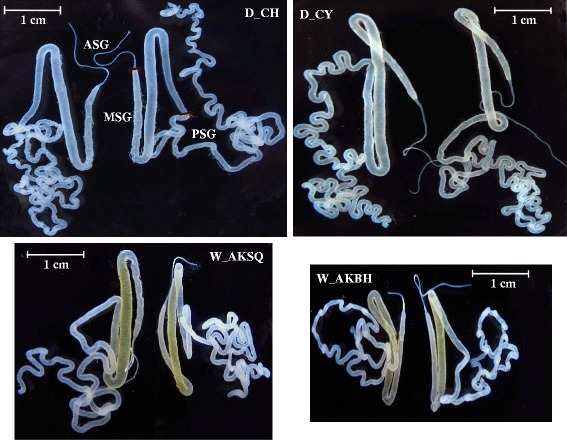
**Anatomy structures of intact silk glands from the domestic and wild silkworms.** The short red lines indicate the boundary regions among the anterior silk gland (ASG), median silk gland (MSG), and posterior silk gland (PSG).

To explore differences in the silk gland transcriptomes between domestic and wild silkworms, two domestic strains, Chunhua (D_CH) and Chunyu (D_CY), and two wild silkworms were selected for analysis. The wild silkworms W_AKBH and W_AKSQ were collected from the Baihe and Shiquan counties of Ankang City, Shaanxi Province, respectively. Intact silk glands were dissected on day 3 of fifth-instar larvae and were found to present similar anatomy structures in the four silkworm (Figure 1). For each silkworm, the RNA from the median/posterior silk glands (M/PSGs) from five female and five male larvae was sequenced using an Illumina Genome Analyzer (II). An overview of the sequencing and assembly is outlined in Table 1, Additional file 1: Table S1. After quality control, the number of clean bases in the D_CH, D_CY, W_AKBH and W_AKSQ transcriptome libraries were 5.48 Gb, 5.74 Gb, 5.2 Gb and 6.06 Gb, respectively (Table 1). We mapped the clean reads to the *B. mori* reference genome, release_2.0 [14]. The proportion of total reads in the four silkworm transcriptome libraries that mapped to the genome ranged from 59.29% to 71.35% (Additional file 1: Table S1).

**Table 1:** **Summary of the sequence assembly after Illumina sequencing**

**Sample name**	**Raw reads**	**Clean reads**	**Clean bases (Gb)**	**Error rate (%)**	**Q20 (%)**	**Q30 (%)**	**GC content (%)**
D_CH_1	28689935	27351142	2.74	0.04	97.24	91.56	50.89
D_CH_2	28689935	27351142	2.74	0.06	94.32	86.63	50.98
D_CY_1	29857247	28748042	2.87	0.03	97.66	92.56	48.77
D_CY_2	29857247	28748042	2.87	0.05	95.17	88.2	48.87
W_AKBH_1	27042449	25978481	2.6	0.04	96.9	90.75	52.46
W_AKBH_2	27042449	25978481	2.6	0.06	94.03	86.19	52.96
W_AKSQ_1	31530647	30344925	3.03	0.03	97.66	92.52	49.08
W_AKSQ_2	31530647	30344925	3.03	0.04	95.53	88.91	49.17

The numbers 1 and 2 at the end of the sample name represent left and right ends (pair-end sequencing), respectively. Gb: Giga base; Q20: percentage of bases with a Phred value of at least 20. Q30: percentage of bases with a Phred value of at least 30.

All the mapped reads from the four silkworms were merged and assembled by Cufflinks [15]. The known silkworm gene models from SilkDB (http://www.silkdb.org/silkdb/) were corrected and novel transcripts were characterized using Cuffcompare. A total of 1,288 new transcripts were detected (Additional file 2: Table S2). Locations of exons and introns from each novel gene are also defined. The 1,288 new transcripts was detected by BLASTN against the silkworm transcriptome database (SilkTransDB), which included transcriptomes from eggs, ant worm and whole body at different developmental stages [16]. A total of 1,074 genes could be found corresponding transcripts in SilkTransDB (Additional file 2: Table S2). All the new transcripts were used to do BLASTX search against the protein non-redundant (nr) database (http://www.ncbi.nlm.nih.gov/). Only 264 genes had no corresponding homologs in nr database (Additional file 3: Table S3). We analyzed the Gene Ontology (GO) classifications of all the 1,288 novel genes (Additional file 4: Figure S1). A total of 584 genes were assigned to corresponding GO terms.

### Transcriptome profiles of the silk glands from four silkworms

**Figure 2:**
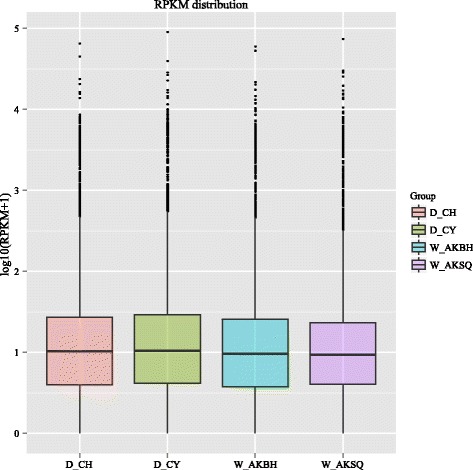
**Boxplot of the log transformed RPKM expression values across four silkworms.** RPKM: Reads per kilo bases per million reads. The solid horizontal line represents the median, and the box encompasses the lower and upper quartiles.

The abundance of all the genes (15,911) was normalized and calculated by the reads per kilo bases per million reads (RPKMs) method using uniquely mapped reads (Additional file 3: Table S3) [17]. Genes with RPKMs in the interval 0–1 were considered not to be expressed or to be present at very low levels; genes with RPKMs over 60 were considered to be expressed at a very high level. The distributions of the expression levels of all the genes were similar for all four silkworms (Figure 2; Table 2). We found that about 58% of the total number of genes (15,911) were expressed (RPKM ≥ 1), and more than 1,118 genes were highly expressed (RPKM > 60) in each silkworm (Table 2). We analyzed the correlation between the topology and biological function of the highly expressed genes using the GO classifications (Additional file 5: Figure S2). In the molecular function category, the most abundant GO terms were “binding” (~40%) and “catalytic activity” (~40%). We found that eight genes, *BGIBMGA009393*, *BGIBMGA005111*, *BGIBMGA001793*, *BGIBMGA011901*, *BGIBMGA003608*, *BGIBMGA001347* and *BGIBMGA013157*, were extremely highly expressed (RPKM > 10,000) in the silk glands of all four silkworms. These genes either encode silk proteins or play roles in the synthesis of proteins. For example, *BGIBMGA005111*, *BGIBMGA009393*, *BGIBMGA001347* and *BGIBMGA011901* correspond to *Fib-H*, *Fib-L*, *P25* and *Ser2*. These results are consistent with the efficient biosynthesis of silk proteins in the silk glands.

**Table 2:** **Distribution of gene expressions in the domestic and wild silkworms**

**RPKM interval**	**D_CH**	**D_CY**	**W_AKBH**	**W_AKSQ**
0–1	6772 (42.56%)	6658 (41.85%)	6804 (42.76%)	6685 (42.01%)
1–3	1568 (9.85%)	1582 (9.94%)	1661 (10.44%)	1632 (10.26%)
3–15	3747 (23.55%)	3726 (23.42%)	3827 (24.05%)	4094 (25.73%)
15–60	2573 (16.17%)	2553 (16.05%)	2371 (14.90%)	2382 (14.97%)
> 60	1251 (7.86%)	1392 (8.75%)	1248 (7.84%)	1118 (7.03%)

RPKM: Reads per kilo bases per million reads. Ratios of gene number to total gene number are presented in parentheses.

### Differentially expressed genes in the silk glands

**Figure 3:**
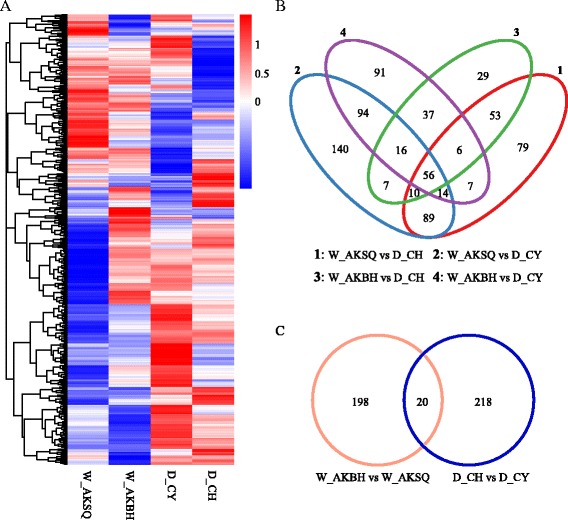
**Hierarchical clustering and Venn diagram of the differentially expressed genes in the silk glands. (A)** Hierarchical clustering of the differentially expressed genes, using the RNA-seq data derived from the silk glands of four silkworms based on log_10_ RPKM values. The blue bands indicate low gene expression quantity; the red bands indicate high gene expression quantity. **(B)** Venn diagram showing the overlaps between the differentially expressed genes (DEGs) in the domestic and wild silkworms. **(C)** Venn diagram of the DEGs in the domestic D_CH vs D_CY and and in the wild W_AKBH vs W_AKSQ.

To identify the DEGs among the four silkworms, the read counts were adjusted using the edgeR program [18] with one scaling normalized factor. DEGs between any two silkworms were identified using the DEGSeq R package [19]. A corrected P-value of 0.005 and log_2_ fold-change of ±1 were set as the threshold for significant differential expression. All the DEGs are presented in Additional file 6: Table S4. To observe the gene expression patterns, we performed hierarchical clustering of all the DEGs based on the log_10_ RPKMs for the four silkworms (Figure 3A).

The distributions of the numbers of DEGs across the four silkworms are shown in Figure 3B and C. We found 238 DEGs between two domestic strains (D_CH and D_CY), and 218 DEGs between the wild silkworms (W_AKBH and W_AKSQ). Only 20 genes showed common differential expressions in comparisons of D_CH vs D_CY and W_AKBH vs W_AKSQ (Figure 3C), implying that most of DEGs between the two domestic silkworm strains were different from the DEGs between the two wild silkworms. As shown in Figure 3B, the numbers of DEGs between the domestic and wild silkworms ranged from 214 to 426. The 56 overlapped genes might be the candidate DEGs in domestic versus wild silkworms (Figure 3B). However, some of the 56 genes showed expression differences within the domestic silkworms or within the wild silkworms and were excluded from the further analysis. Thus, the remaining 32 genes were selected as the major DEGs in domestic versus wild silkworms.

**Figure 4:**
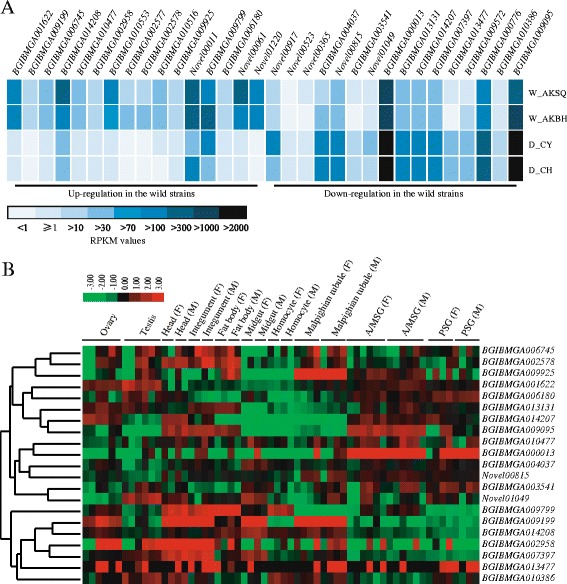
**Expression patterns of differentially expressed genes in domestic versus wild silkworms. (A)** Expression levels of the differentially expressed genes (DEGs) in four silkworm strains. **(B)** Tissue expression patterns of the DEGs in different larvae tissues based on microarray data [11]. Twenty-one of the 32 DEGs in domestic versus wild silkworms had probes in the microarray data (Table 3). Hierarchical clustering was performed using Cluster software (http://genome-www.stanford.edu/clustering/) with the average linkage method. F: female; M: male. A/MSG: anterior/median silk gland; PSG: posterior silk gland.

Based on the transcriptome expression data (Additional file 3: Table S3), the expression patterns of the 32 candidate DEGs in wild versus domestic silkworms are presented in Figure 4A. Sixteen of these genes were up-regulated in the domestic silkworms and 16 were up-regulated in the wild silkworms. Four genes, *BGIBMGA009199*, *BGIBMGA002577*, *BGIBMGA002578*, and *Novel01220*, showed no or very low expressions (RPKM < 1) in the domestic silkworms, while *Novel00523* had no or very low expression in the wild silkworms (Figure 4A). Based on the genome-wide microarray data of the domestic silkworm published previously [11], we surveyed the expressions of the 32 DEGs in multiple tissues from day 3 of fifth-instar larvae (Figure 4B) and found that most of these DEGs were expressed in other tissues as well as in the silk glands (Figure 4B).

### GO and KEGG enrichment analyses of the differentially expressed genes

A total of 15,911 unique genes matched known proteins in the NCBI nr database and in InterPro (http://www.ebi.ac.uk/interpro/) by BLAST searches and 6555 (41.2%) of these genes were annotated with GO terms. The GO terms were converted to the generic GO Slim terms using Blast2GO [20]. To analyze the gene functions of the DEGs, a GO enrichment analysis was performed using Fisher’s exact test in Blast2GO. GO terms with corrected P-value < 0.05 were considered significantly enriched among the DEGs.

The GO functional enrichment analysis of the 218 DEGs between the two wild silkworms revealed significantly enriched terms in the biological process and molecular function categories (Additional file 7: Table S5). Metabolic process (GO: 0008152) with 51 genes was dominant in the biological process category and protein binding (GO: 0005515) with six genes was dominant in molecular function category. In the analysis of the 238 DEGs between the domestic silkworm strains, oxidoreductase activity (GO: 0016491) with 31 genes was the only significantly enriched term. These results suggested that there may be more differences in protein synthesis events between wild silkworms than between domestic silkworms. Oxidoreductase activity was the only commonly enriched term related to the DEGs in four pairwise comparisons between the wild and domestic silkworms (Additional file 7: Table S5).

**Figure 5:**
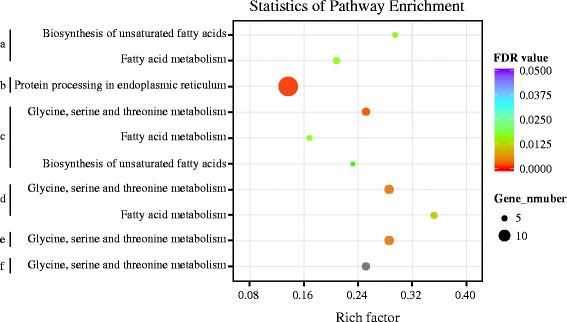
**Scatterplot of enriched KEGG pathways for differentially expressed genes between any two silkworms.** Rich factor is the ratio of the differentially expressed gene number to the total gene number in a certain pathway. The characters a, b, c, d, e, and f correspond to the comparisons D_CH vs D_CY, W_AKSQ vs W_AKBH, W_AKBH vs D_CH, W_AKBH vs D_CY, W_AKSQ vs D_CH, and W_AKSQ vs D_CY, respectively. The size and color of the dots represent the gene number and the range of the FDR value, respectively. The grey dot indicates a pathway that was not significantly enriched (the FDR value is 0.08).

KEGG pathway enrichment analysis was done using KOBAS 2.0 [21]. We found that the protein processing in endoplasmic reticulum pathway was significantly enriched (false discovery rate < 0.05) for the DEGs between the two wild silkworms (Figure 5). Two over-represented pathways, biosynthesis of unsaturated fatty acids and fatty acid metabolism, were also identified for the DEGs in the two domestic silkworm strains. In the four pairwise comparisons between the wild and domestic silkworms, except W_AKSQ vs D_CY, the glycine, serine and threonine metabolism pathway was significantly enriched for the DEGs. We also found seven DEGs for W_AKSQ vs D_CY that were related to glycine, serine and threonine metabolism; however, the pathway was not found to be significantly enriched (Figure 5).

Based on the GO and KEGG enrichment analyses, we sorted the 32 candidate DEGs into corresponding functional categories and pathways. *BGIBMGA010516* and *BGIBMGA009925* were predicted to play roles in mediating transformation of serine to glycine in the glycine, serine and threonine metabolism pathway. A previous study revealed that glycine and serine were the major amino acid residues in the silk fibroin heavy chain [7]; thus, the differential expressions of these two genes might affect the amino acid composition of fibroins in wild silkworms. Interestingly, seven of the 16 up-regulated genes in the wild silkworms were assigned the GO term “oxidoreductase activity”, which is associated with antioxidant systems. We also manually annotated the 32 DEGs and proposed functions based on their homologous proteins identified in BLAST searches (Table 3). Five of the up-regulated DEGs in the wild silkworms were found to be involved in immune response, suggesting that these DEGs may play roles in the silkworms’ response to pathogens and environmental conditions. Some of the up-regulated DEGs in domestic silkworms were predicted to be related to tissue development (*Novel0104*9), excretion of proteins (*BGIBMGA013477* and *BGIBMGA009572*), and metabolism (*BGIBMGA013131* and *BGIBMGA014207*) (Table 3), suggesting that these DEGs may be involved in the highly efficient biosynthesis and secretion of silk proteins.

**Table 3:** **Functional annotation of the differentially expressed genes in domestic versus wild silkworms**

**Gene name**	**Best BLAST hit in nr database; putative function**	**E-value**	**Microarray probe**
*BGIBMGA001622*	Uncharacterized protein	6E-54	sw21843
*BGIBMGA009199*	Organic cation transporter protein-like; excretion of endogenous compounds and xenobiotics [22]	0.0	sw15503
*BGIBMGA006745* ^*^	laccase 2A; immune response and/or detoxification [23,24]	0.0	sw12993
*BGIBMGA014208*	Cytochrome b5; electron transfer component in a number of oxidative reactions [25]	2E-84	sw03099
*BGIBMGA010477*	Scavenger receptor class B member 4; absorption of carotenoid [26]	0.0	sw01562
*BGIBMGA002958*	Dopa decarboxylase 2; immune response [27]	0.0	sw15376
*BGIBMGA010553*	Putative fatty acyl-CoA reductase	2E-58	
*BGIBMGA002577* ^*^	Peroxisomal acyl-coenzyme A oxidase 1-like	0.0	
*BGIBMGA002578* ^*^	Peroxisomal acyl-coenzyme A oxidase 1-like	0.0	sw22703
*BGIBMGA010516* ^*^	Glucose dehydrogenase [acceptor]-like	0.0	
*BGIBMGA009925* ^*^	Glucose dehydrogenase [acceptor]-like	0.0	sw21310
*Novel00011*	No hits		
*BGIBMGA009799* ^*^	Aldose reductase-like isoform X1	0.0	sw19860
*BGIBMGA006180* ^*^	Venom dipeptidyl peptidase 4-like isoform X2; immune response [28]	0.0	sw14578
*Novel00061*	No hits		
*Novel01220*	Serine protease inhibitor 16 precursor; immune response [29]	3E-37	
***Novel00917***	No hits		
***Novel00523***	Uncharacterized protein	2E-63	
***Novel00365***	No hits		
***BGIBMGA004037***	Hypothetical protein EAG_07492	1E-10	sw04615
***Novel00815***	Putative pol-like protein	4E-52	sw21903
***BGIBMGA003541***	Hypothetical protein KGM_18541	3E-169	sw22611
***Novel01049***	Ubiquitin-like modifier-activating enzyme 5; to activate and transfer ubiquitin to ubiquitin conjugating enzymes [30]	0.0	sw12010
***BGIBMGA000013***	Osiris 9A	1E-96	sw13441
***BGIBMGA013131***	Apolipoprotein D-like; transporting lipids and other small hydrophobic molecules for metabolism [31]	1E-154	sw15906
***BGIBMGA014207***	Cytochrome b5; electron transfer component in a number of oxidative reactions [25]	3E-90	sw08853
***BGIBMGA007397***	Protein charybde-like	8E-78	sw03416
***BGIBMGA013477***	Synaptic vesicle glycoprotein 2B-like isoform X1; regulating excretion of proteins [32,33]	0.0	sw21450
***BGIBMGA009572***	Protein translocase subunit secA; protein export[34]	0.0	
***BGIBMGA000776***	Juvenile hormone esterase-like	0.0	
***BGIBMGA010386***	Non-lysosomal glucosylceramidase-like; glucosylceramide degradation pathway [35]	0.0	sw07262
***BGIBMGA009095***	Fungal protease inhibitor F-like isoform X1; immune response [29]	2E-53	sw01309

Up-regulated genes in the domestic strains are shown in bold; up-regulated genes in the wild silkworms are in normal font. ^*^Genes related to oxidoreductase activity in the GO molecular function category. The microarray probes are from Xia et al. [11].

### Validation of RNA-seq data by quantitative real-time PCR

**Figure 6:**
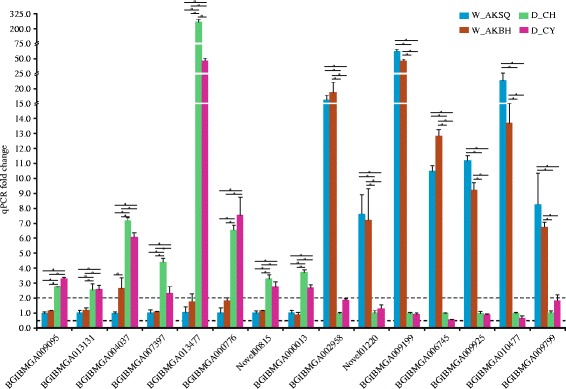
**Quantitative real-time PCR validation of the differentially expressed genes.** The relative expression of a candidate gene was normalized against *RpL3*. For the up-regulated DEGs in the domestic silkworms, the fold-change of each gene was calculated by dividing the relative expression level in the W_AKSQ. For the up-regulated DEGs in the wild silkworms, the fold-change of each gene was calculated by dividing the relative expression level in the D_CH. The data are the average ± standard error of three independent replicated qPCR experiments. An absolute value of fold-change ≥ 2 and one-way analysis of variance analysis (P-value < 0.005) were used to estimate the significance of gene expression changes. Significant differential expressions of genes between any two silkworms were marked by a star.

To validate the RNA-seq data, qPCR was performed for 15 DEGs that we randomly selected from domestic versus wild silkworms transcriptome data. The selected genes were found to show differential expressions between the domestic and wild silkworms, and no significant difference between the two wild silkworms or two domestic silkworms. As shown in Figure 6 and Additional file 8: Table S6, the qPCR expression results were similar to the results obtained from the Illumina sequencing data (Additional file 6: Table S4). That is, the fold changes in gene expression determined by qPCR were basically consistent with their transcript abundance changes determined by RNA-seq, which confirmed the validity of the expression data. Only two of the 15 selected DEGs showed significant expression differences (absolute fold-change ≥ 2 and P-value < 0.005) between the two wild silkworms (BGIBMGA004037, fold-change = 2.7) and between the two domestic silkworm strains (BGIBMGA013477, fold-change = 5.6).

## Discussion

The cocoons of domestic silkworms are much larger than those of wild silkworms [4] and the silk production of domestic silkworms is nearly 3-fold that of wild silkworms. For example, the cocoon shell weights of W_AKBH, W_AKSQ, D_CH and D_CY were 0.13 g, 0.12 g, 0.32 g and 0.36 g, respectively. Characterization of a transcriptome can help explain the functional complexity of a genome and reveal cell activities like growth, development and immune response. Here, we compared the silk gland transcriptomes from the wild and domestic silkworms with the aim of obtaining useful information for understanding the biosynthesis and secretion of the silk proteins.

To help understand protein synthesis and development in the silk gland, DEGs in domestic versus wild silkworms were identified (Additional file 6: Table S4). We characterized 32 DEGs, 16 of which were up-regulated in domestic silkworms (Figure 4A). We manually annotated the functions of these 16 DEGs, and found that some of them were related to tissue development, excretion of proteins, and metabolism (Table 3). For example, we predicted that *Novel01049* might encode an ubiquitin-activating enzyme that could initiate the ubiquitination of proteins [30]. Ubiquitination is an essential process that regulates the turnover of proteins for basic cellular processes such as the cell cycle and cell death [30]. *BGIBMGA013477* was predicted to encode synaptic vesicle protein 2B (SV2B), which is a vesicle protein present in the secretory vesicles and is an important regulator of Ca^2+^-stimulated vesicle exocytosis [32]. Secretory vesicles have also been found in the silk gland of the domestic silkworm [36]. *BGIBMGA013477* (SV2B) was up-regulated more than 256-fold in D_CH and 46-fold in D_CY compared with its expression in the wild silkworms (Figure 6). SV2B might mediate the release of neurotransmitter and regulate secretion of silk proteins in the silk gland. *BGIBMGA009572* was predicted to encode translocase subunit secA, which might play role in pushing substrates forward like a “motor” [34]. The Sec translocase pathway is ubiquitous and is responsible for the vast majority of protein export activities [34]. Thus, the up-regulated DEGs in silk glands of the domestic silkworms might be associated with the highly efficient biosynthesis and secretion of the silk proteins.

Fibroins have been found to be the major components (75%) of silk threads [6]. In the present study, we found that the *Fib-H*, *Fib-L*, and *P25* were extremely highly expressed and showed similar expression levels in both the domestic and wild silkworms (Additional file 3: Table S3). Initially, it was difficult to understand the obvious differences in silk production between domestic and wild silkworms; however, by combining our results with previously reported findings, we propose two possible explanations for this anomaly. In the transcriptome analysis, we concentrated only on protein-coding genes, while it has been reported that microRNAs (miRNAs) may play regulatory functions in the silk protein synthesis [37]. MiRNAs play important roles in a broad range of biological processes, including down-regulating the translation of target genes to proteins [38], inhibiting translation initiation, and mediating mRNA decay [39]. A total of 1,229 miRNAs have been identified in the PSGs in fifth-instar larvae of the silkworm using next-generation sequencing and microarray assay [37] and the fibroin encoding genes *Fib-H*, *Fib-L*, and *P25* have been reported to be the targets of some miRNAs [37]. Because we found that the expression levels of the fibroin genes were similar in domestic and wild silkworms, we propose that miRNAs might be involved in the down-regulation of fibroin synthesis in the wild silkworms. Another possible explanation is, because silk fibroins are synthesized in PSG cells then secreted into the PSG lumen and aggregated in the MSG [40], if the synthesized silk proteins are not secreted rapidly, their biosynthesis might be inhibited by the accumulated silk proteins. In the domestic silkworms, the up-regulated genes related to secretion (Table 3) might promote the secretion of silk proteins for the continued synthesis of new proteins. Thus, highly efficient biosynthesis and secretion may be associated with higher silk production in the domestic silkworms.

Sixteen up-regulated DEGs were found in the wild silkworms compared with the domestic silkworms; seven of them were related to the GO term “oxidoreductase activity” (Additional file 7: Table S5) and a further six were related to immune response or detoxification (Table 3). For example, laccase (*BGIBMGA006745*) and dopa decarboxylase (*BGIBMGA002958*) take part in the melanization cascade, which has been proposed to play roles in the immune response of insects [23,27,41]. In *B. mori*, serine protease inhibitor 16 (*Novel01220*) may be involved in resistance to pathogenic microorganisms [29]. In *Manduca sexta*, laccase may play an important role in the oxidation of toxic compounds in the diet [24]. Interestingly, *BGIBMGA009199* is thought to be associated with domestication [2], and we found that it was up-regulated more than 46-fold in the wild silkworms (Figure 6; Additional file 8: Table S6). *BGIBMGA009199* encodes an organic cation transporter, which might mediate the transport of a variety of endogenous compounds and numerous drugs and xenobiotics [22]. Wild silkworms live in a more natural niche than domestic silkworms and therefore are likely to encounter more xenobiotics and pathogenic microorganisms than domestic silkworms. Thus, the up-regulated DEGs in the wild silkworms may play important roles in dealing with xenobitics and pathogens in the natural environment.

## Conclusions

In summary, this study represents a significant step in the characterization of silk gland transcriptomes and provides insights into the molecular mechanisms of silk production. The transcriptome comparisons revealed that DEGs associated with immune response and detoxification were up-regulated in the wild silkworms, which is consistent with their exposure to more pathogens and xenobitics in the natural environment than the domestic silkworms. In the domestic silkworms, DEGs that may be associated with highly efficient biosynthesis and secretion of silk proteins were up-regulated. However, further research is required to determine whether these DEGs are the genes responsible for the difference in silk production between domestic and wild silkworms. Further functional exploration of these genes may provide evidence for their future application in sericulture.

## Methods

### Silkworm collection and sample preparation

Wild silkworms W_AKBH and W_AKSQ were collected from the Baihe and Shiquan counties of Ankang City, in Shaanxi Province, respectively. The larvae were reared on live mulberry trees in open-air cages. Domestic silkworm strains Chunhua (D_CH) and Chunyu (D_CY) were reared on mulberry leaves under stable 14 h light and 10 h dark photoperiod at 25 ± 1°C and 75% ± 3% relative humidity. Intact silk glands were dissected on day 3 of fifth-instar larvae. The ASG was removed because the ASG is the tube that is used for spinning the silk. For each of the four silkworms, the M/PSGs from five male larvae and five female larvae were pooled and used as one sample. All the samples were frozen immediately in liquid nitrogen until use.

### RNA extraction, library preparation and sequencing

Total RNA was extracted using TRIzol reagent according to the manufacturer’s protocol (Invitrogen, Burlington, ON, Canada). RNA degradation and contamination was monitored on 1% agarose gels. RNA purity was checked using a Nano Photometer spectrophotometer (Implen, Westlake Village, CA). RNA integrity was assessed using the RNA 6000 Nano Assay Kit with a Bioanalyzer 2100 (Agilent Technologies, Santa Clara, CA) after checking the RNA purity and concentration.

A total of 3 μg RNA per sample was used as input material for RNA sample preparations. The transcriptome libraries were generated using Illumina TruSeq™ RNA Sample Preparation Kit (Illumina, San Diego, CA) following the manufacturer’s recommendations. Clustering of the index-coded samples was performed on a cBot Cluster Generation System using TruSeq PE Cluster Kit v3-cBot-HS (Illumina) according to the manufacturer’s instructions. After clustering, the libraries were sequenced on an Illumina Hiseq 2000 platform and 100-bp paired-end reads were generated.

### Transcriptome data analysis

#### Quality control

Raw data (raw reads) in FASTQ format were processed using in-house Perl scripts to remove reads that contained adapter sequences, reads that contained ploy-N stretches (i.e., partially unsequenced regions), and low quality reads. The Q20, Q30, GC content, and sequence duplication level of the resultant clean sequences were calculated. All the downstream analyses are based on the clean sequence with high quality.

#### Mapping and assembly of clean reads

The reference genome and gene model annotation files were downloaded from the Silkworm Genome Database (SilkDB; http://www.silkdb.org/silkdb/). An index of the reference genome was built using Bowtie v2.0.6 [42] and paired-end clean reads were aligned to the reference genome using TopHat v2.0.7 [43]. The Reference Annotation Based Transcript (RABT) assembly method implemented in Cufflinks v1.3.0 [15] was used to construct and identify known and novel transcript fragments from the TopHat alignment results.

#### Quantification and differential expression analysis of transcripts

HTSeq v0.5.3 (http://www-huber.embl.de/users/anders/HTSeq) was used to count the number of reads mapped to each transcript and RPKM was used to quantify transcripts expression. RPKM was calculated based on the mapped transcript fragments, transcript length and sequencing depth.

The read counts were adjusted using the edgeR Bioconductor [18] with one scaling normalized factor prior to differential gene expression analysis, which was performed using the DEGSeq R package, release 1.12.0 (TNLIST, Beijing, China). The P-values were adjusted for multiple testing using the Benjamini-Hochberg method [44]. A corrected P-value of 0.005 and log_2_ fold-change of ±1 were set as the threshold for significant differential expression.

#### GO annotation and GO/KEGG enrichment analyses

All the genes were annotated for protein function using InterProScan (http://www.ebi.ac.uk/interpro/) and BLASTX against the NCBI nr database. The resulting InterPro and BLAST annotations were converted into GO annotations and All the GO terms were mapped to the GO Slim categories. The statistical significance of the functional GO Slim enrichment was evaluated using the Fisher’s exact test within Blast2GO (false discovery rate (FDR) < 0.05) [20]. Significantly enriched KEGG pathways were identified with KOBAS 2.0 [21] using a hypergeometric test and the Benjamini-Hochberg FDR correction.

### Validation of differentially expressed genes by quantitative real-time PCR

We used the mapping algorithm in the CLC Genomics Workbench v6.5.1 (CLC bio, Cambridge, MA) to map the reads to the assembled coding sequences. Conservative parameters (mismatch, insertion and deletion cost of 3, and length fraction and similarity fraction of 0.9) were set to prevent mis-mapping of paralogous sequences. All the read-mappings were inspected by eye. The validated gene sequences in the four silkworms were aligned with Clustal X [45]. The primers used for the qPCR were designed based on the consensus sequence of each alignment. qPCR was performed using a CFX96™ Real-Time PCR Detection System (Bio-Rad, Hercules, CA) with SYBR Green qPCR Mix (Bio-Rad). The cycling parameters were as follows: 95°C for 3 min, then 40 cycles of 95°C for 10 s, and annealing for 30 s (the specific annealing temperature for each PCR are listed in Additional file 9: Table S7). Each sample was analyzed in triplicate, and gene expression levels were normalized against the corresponding ribosomal protein L3 (*RpL3*) or α-tubulin expression levels. The relative expression levels were analyzed using the classic R = 2^-ΔΔCt^ method [46].

### Availability of supporting data

Raw reads of transcriptome have been deposited into the NCBI Short Read Archive (SRA, http://www.ncbi.nlm.nih.gov/sra/) under the accession numbers SRR1592681, SRR1592710, SRR1592737, and SRR1592738.

## Additional files

Additional file 1: Table S1. Summary of RNA-seq data mapped to silkworm reference genome. Additional file 2: Table S2. Gene structure predictions and sequences of the novel transcripts. The 1288 novel transcripts were detected by BLASTN searches against the silkworm transcriptome database (SilkTransDB, http://124.17.27.136/gbrowse2/). For novel genes that shared identity ≥ 99% with transcripts in SilkTransDB with E value ≤ 1e-30, the corresponding SilkTransDB transcript is shown. For the other novel gene, there is a slash in the corresponding row. Additional file 3: Table S3. Expression levels of all the genes across the four silkworms. A BLAST search was conducted using all the 15,911 unique transcripts as query sequences against the protein non-redundant (nr) database (http://www.ncbi.nlm.nih.gov/). The best BLAST hits and E-values are listed. Additional file 4: Figure S1. Gene Ontology (GO) classification of the novel genes identified in this study. The 584 annotated novel genes were classified into the three GO functional categories: molecular function, biological process and cellular component. Additional file 5: Figure S2. Gene Ontology classification of highly expressed genes in the silk gland of four silkworms. Genes with RPKM greater than 60 were considered to be highly expressed. Additional file 6: Table S4. Differentially expressed genes between any two silkworm samples. Log_2_ (FC): log_2_ fold-change. The P-values were adjusted for multiple testing using the Benjamini-Hochberg method [44]. An adjusted P-value (q-value) of 0.005 and log_2_ fold-change of ±1 were set as the threshold for significant differential expression. Additional file 7: Table S5. Gene Ontology enrichment of the DEGs between any two silkworms. Additional file 8: Table S6. Fold-changes of the differentially expressed genes validated by qPCR. The data are the average ± standard error of three independent replicated qPCR experiments. Additional file 9: Table S7. Primer sequences used for the qPCR validation experiment. The accession numbers of *RpL3* and α-tubulin are NM_001043661 and NM_001043419 in GenBank, respectively. The accession numbers beginning with BGI are from SilkDB (http://www.silkdb.org/silkdb/).

## Abbreviations

RPKM: Reads Per Kilo bases per Million reads
DEGs: Differentially expressed genes
M/PSG: median/posterior silk gland
GO: Gene Ontology
KEGG: Kyoto Encyclopedia of Genes and Genomes
