# Supplementary material for: Comparative analysis of the silk gland transcriptomes between the domestic and wild silkworms
Source: BMC Genomics. 2015 Feb 6;16(1):60. doi: 10.1186/s12864-015-1287-9 (PMC4328555; doi:10.1186/s12864-015-1287-9)
Supplement: Additional file 1: Table S1. — Summary of RNA-seq data mapped to silkworm reference genome. [file 12864_2015_1287_MOESM1_ESM.docx]

**Additional file 1: Table S1**

| Sample name | D_CH | D_CY | W_AKBH | W_AKSQ |
| --- | --- | --- | --- | --- |
| Total clean reads | 54702284 | 57496084 | 51956962 | 60689850 |
| Total mapped | 34843587 (63.7%) | 41026317 (71.35%) | 30804346 (59.29%) | 40259708 (66.34%) |
| Multiple mapped | 1775089 (3.24%) | 1927146 (3.35%) | 1384534 (2.66%) | 2187501 (3.6%) |
| Uniquely mapped | 33068498 (60.45%) | 39099171 (68%) | 29419812 (56.62%) | 38072207 (62.73%) |
| Read-1 | 16754837 (30.63%) | 19769613 (34.38%) | 14882110 (28.64%) | 19245989 (31.71%) |
| Read-2 | 16313661 (29.82%) | 19329558 (33.62%) | 14537702 (27.98%) | 18826218 (31.02%) |
| Reads map to '+' | 16340300 (29.87%) | 19435525 (33.8%) | 14496485 (27.9%) | 18899170 (31.14%) |
| Reads map to '-' | 16728198 (30.58%) | 19663646 (34.2%) | 14923327 (28.72%) | 19173037 (31.59%) |
| Non-splice reads | 22822699 (41.72%) | 26031455 (45.28%) | 19697576 (37.91%) | 25944213 (42.75%) |
| Splice reads | 10245799 (18.73%) | 13067716 (22.73%) | 9722236 (18.71%) | 12127994 (19.98%) |
| Reads mapped in proper pairs | 25799696 (47.16%) | 31155246 (54.19%) | 21705578 (41.78%) | 28680324 (47.26%) |
| Proper-paired reads map to different chromosome | 10726 (0.02%) | 9444 (0.02%) | 8698 (0.02%) | 15078 (0.02%) |
