# Supplementary figures and images for: Comparative analysis of the silk gland transcriptomes between the domestic and wild silkworms
Source: BMC Genomics. 2015 Feb 6;16(1):60. doi: 10.1186/s12864-015-1287-9 (PMC4328555; doi:10.1186/s12864-015-1287-9)

# WEGO Output

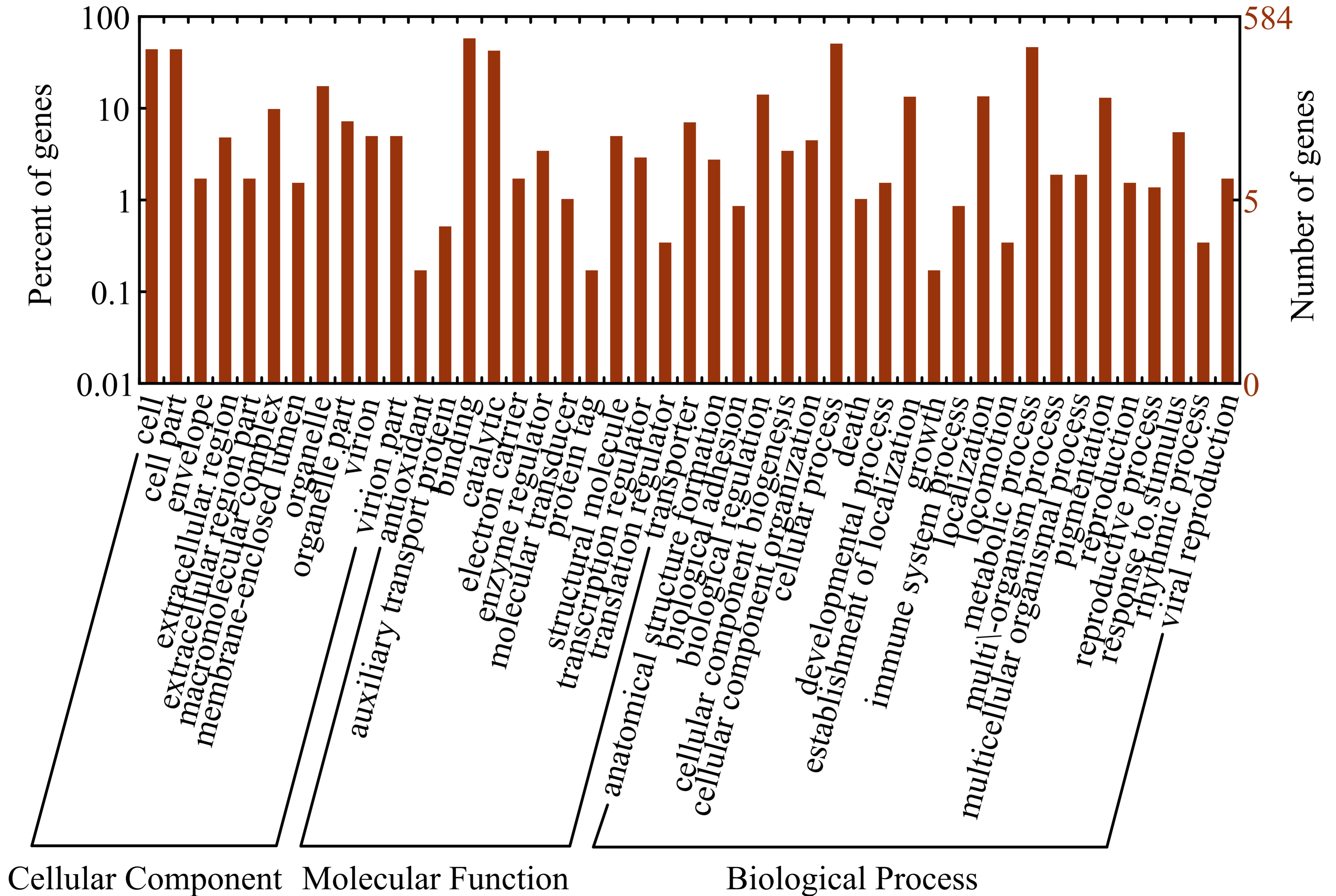

Supplement: Additional file 4: Figure S1. — Gene Ontology (GO) classification of the novel genes identified in this study. The 584 annotated novel genes were classified into the three GO functional categories: molecular function, biological process and cellular component. [file 12864_2015_1287_MOESM4_ESM.pdf]

# WEGO output

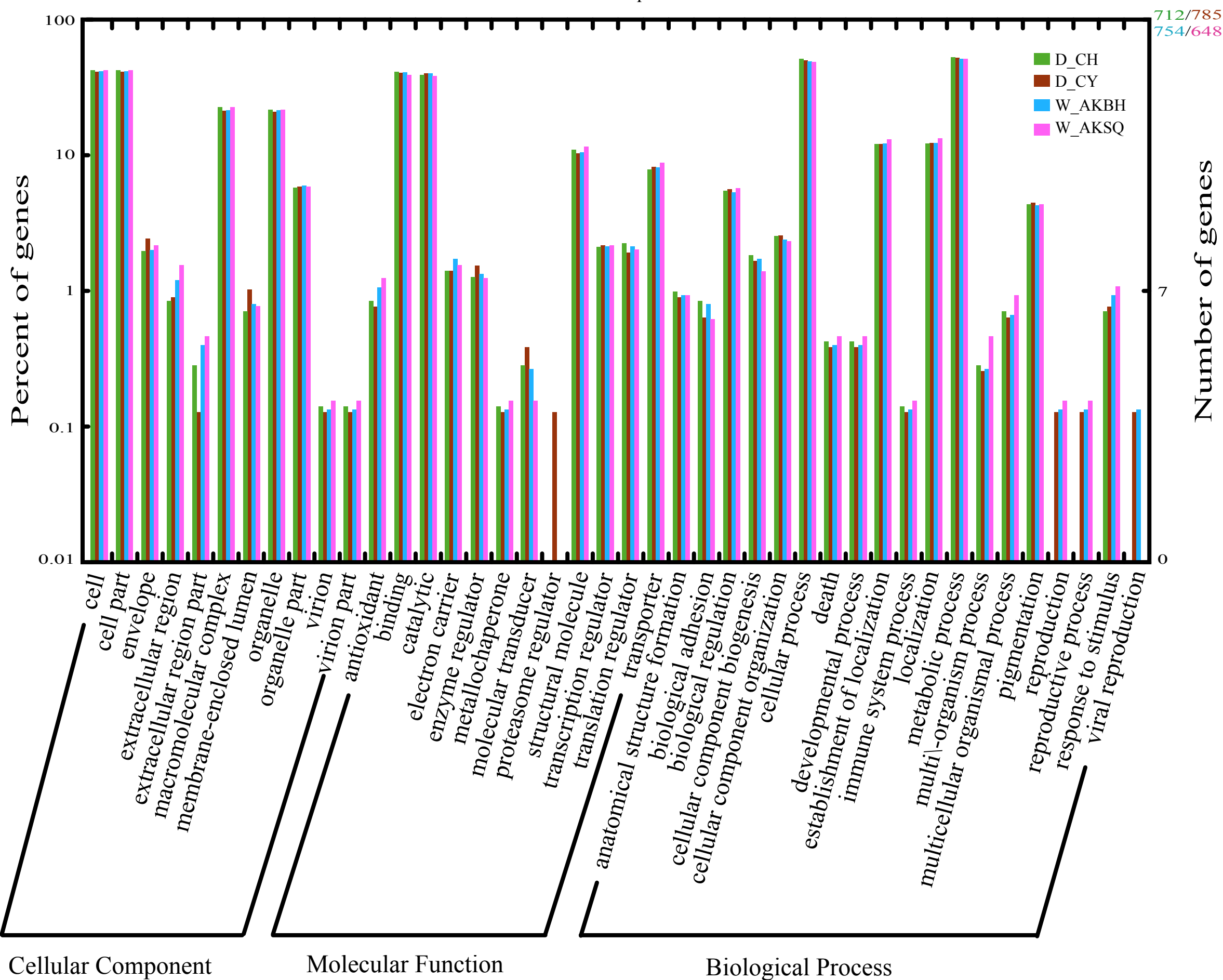

Supplement: Additional file 5: Figure S2. — Gene Ontology classification of highly expressed genes in the silk gland of four silkworms. Genes with RPKM greater than 60 were considered to be highly expressed. [file 12864_2015_1287_MOESM5_ESM.pdf]
