# Supplementary material for: Comparative analysis of the silk gland transcriptomes between the domestic and wild silkworms
Source: BMC Genomics. 2015 Feb 6;16(1):60. doi: 10.1186/s12864-015-1287-9 (PMC4328555; doi:10.1186/s12864-015-1287-9)
Supplement: Additional file 8: Table S6. — Fold-changes of the differentially expressed genes validated by qPCR. The data are the average ± standard error of three independent replicated qPCR experiments. [file 12864_2015_1287_MOESM8_ESM.docx]

**Additional file 8: Table S6**

| **Genes** | **W_AKSQ** | **W_AKBH** | **D_CH** | **D_CY** |
| --- | --- | --- | --- | --- |
| *BGIBMGA009095* | 1.001 ± 0.066 | 1.178 ± 0.011 | 2.742 ± 0.054 | 3.313 ± 0.105 |
| *BGIBMGA013131* | 1.013 ± 0.197 | 1.219 ± 0.148 | 2.560 ± 0.400 | 2.599 ± 0.266 |
| *BGIBMGA004037* | 1.003 ± 0.096 | 2.663 ± 0.712 | 7.188 ± 0.199 | 6.085 ± 0.291 |
| *BGIBMGA007397* | 1.018 ± 0.230 | 1.086 ± 0.018 | 4.421 ± 0.256 | 2.354 ± 0.426 |
| *BGIBMGA013477* | 1.055 ± 0.390 | 1.774 ± 0.525 | 256.808 ± 20.766 | 46.069 ± 4.235 |
| *BGIBMGA000776* | 1.031 ± 0.323 | 1.833 ± 0.190 | 6.550 ± 0.337 | 7.528 ± 1.231 |
| *Novel00815* | 1.005 ± 0.125 | 1.146 ± 0.064 | 3.288 ± 0.267 | 2.738 ± 0.352 |
| *BGIBMGA000013* | 1.013 ± 0.200 | 0.899 ± 0.143 | 3.729 ± 0.178 | 2.684 ± 0.238 |
| *BGIBMGA002958* | 16.387 ± 1.505 | 18.595 ± 3.445 | 1.001 ± 0.063 | 1.883 ± 0.075 |
| *Novel01220* | 7.629 ± 1.288 | 7.221 ± 2.117 | 1.010 ± 0.173 | 1.291 ± 0.272 |
| *BGIBMGA009199* | 62.042 ± 3.784 | 46.787 ± 2.316 | 1.001 ± 0.062 | 0.932 ± 0.087 |
| *BGIBMGA006745* | 10.493 ± 0.374 | 12.848 ± 0.417 | 1.000 ± 0.009 | 0.545 ± 0.046 |
| *BGIBMGA009925* | 11.179 ± 0.367 | 9.231 ± 0.500 | 1.003 ± 0.099 | 0.890 ± 0.071 |
| *BGIBMGA010477* | 22.730 ± 3.328 | 13.711 ± 1.602 | 1.001 ± 0.049 | 0.653 ± 0.170 |
| *BGIBMGA009799* | 8.253 ± 2.109 | 6.742 ± 0.342 | 1.006 ± 0.130 | 1.844 ± 0.375 |
