# Supplementary material for: Comparative analysis of the silk gland transcriptomes between the domestic and wild silkworms
Source: BMC Genomics. 2015 Feb 6;16(1):60. doi: 10.1186/s12864-015-1287-9 (PMC4328555; doi:10.1186/s12864-015-1287-9)
Supplement: Additional file 9: Table S7. — Primer sequences used for the qPCR validation experiment. The accession numbers of RpL3 and α-tubulin are NM_001043661 and NM_001043419 in GenBank, respectively. The accession numbers beginning with BGI are from SilkDB (http://www.silkdb.org/silkdb/). [file 12864_2015_1287_MOESM9_ESM.docx]

**Additional file 9: Table S7**

| Accession number/  gene name | Forward primer (5'-3') | Reverse primer (5'-3') | Length of amplificon (bp) | Anneal temperature (°C) |
| --- | --- | --- | --- | --- |
| *BGIBMGA000013* | GGAATCATTGCCTTTGCCGC | CTCCACCGCCACCGCTACTGT | 178 | 56.3 |
| *BGIBMGA000776* | TCGTATCAGGGTGACATCAGCATT | GGAGAAATGTGGTGGTGCGAAAC | 109 | 52.5 |
| *BGIBMGA002958* | GATCCTTTACATGAATTAGGGCCAA | AATCGAAATTAACCGGTAGCCACTT | 178 | 52.7 |
| *BGIBMGA004037* | CAGGCGGCTAAGGATGCTAT | TGCTGCCCATTGTAGTGTTGC | 212 | 53.6 |
| *BGIBMGA006745* | AGACAACCGACCCGCTAACT | GCAAGTGGAACGGATGAGATA | 134 | 51.4 |
| *BGIBMGA007397* | CGCACGAAATACATCTACACCTTGA | ACTCCGCCTGGCTCCTGAAC | 153 | 54.6 |
| *BGIBMGA009095* | TGCTCACACGACATTGAACCC | TCCGAGTTGAAGAGGTATCCAGAAT | 123 | 54.6 |
| *BGIBMGA009199* | CGATGGACAGGTTCGGGAGG | GATCATGTTGAGCGTCGTTTGG | 125 | 54.6 |
| *BGIBMGA009799* | GACGATCAGGCCGCTGTTCT | CGCCGTTGACTCCATTCTTC | 119 | 54.4 |
| *BGIBMGA009925* | TGGCTCCTCACTGGATTGGC | CACCCTGAGATGTTCATTCTGTCG | 180 | 53.1 |
| *BGIBMGA010477* | CGTGATAGCGTTGCTGGTGA | CTTCGTCTTTGGGCTTCTCG | 104 | 56.0 |
| *BGIBMGA013131* | TGTCCCATTGTTGAGCCTAT | GTCGGTCCTGCTAAAGTTGT | 120 | 50.7 |
| *BGIBMGA013477* | CCACCAAGAATGACTTCGAGCTGT | GAGCGTCCGCCAAGTTACCC | 111 | 57.9 |
| *Novel00815* | GAGAAGACAGGGCTGATGGA | CAAGGAGGGATGAGGGACA | 164 | 51.2 |
| *Novel01220* | GACAACGAGGATCTTAGCG | AGCCCACAGAAGAGCGTAA | 182 | 51.1 |
| RpL3 | CGGTGTTGTTGGATACATTGAG | GCTCATCCTGCCATTTCTTACT | 161 | 55 |
| α-tubulin | ACATGGCTTGCTGTATGCT | GGGTGGCTGGTAGTTGATA | 146 | 55.5 |

Note: The accession numbers of RpL3 and α-tubulin are NM_001043661 and NM_001043419 in GenBank, respectively. The accession numbers beginning with BGI are from SilkDB (<http://www.silkdb.org/silkdb/>).
